# Oral bisphosphonates do not increase the risk of severe upper gastrointestinal complications: a nested case–control study

**DOI:** 10.1186/1471-230X-14-5

**Published:** 2014-01-07

**Authors:** Arianna Ghirardi, Lorenza Scotti, Gianluca Della Vedova, Luca Cavalieri D’Oro, Francesco Lapi, Francesco Cipriani, Achille P Caputi, Alberto Vaccheri, Dario Gregori, Rosaria Gesuita, Annarita Vestri, Tommaso Staniscia, Giampiero Mazzaglia, Giovanni Corrao

**Affiliations:** 1 Department of Statistics and Quantitative Methods, Unit of Biostatistics and Epidemiology, University of Milano-Bicocca, Via Bicocca degli Arcimboldi 8, 20126 Milan, Italy; 2 Department of Informatics, Systems and Communications, University of Milano-Bicocca, Milan, Italy; 3 Operative Unit of Epidemiology, Local Health Unit of Monza and Brianza, Monza, Italy; 4 Department of Epidemiology, Regional Agency for Healthcare Services of Tuscany, Florence, Italy; 5 Department of Preclinical and Clinical Pharmacology, University of Florence, Florence, Italy; 6 Centre for Clinical Epidemiology, Jewish General Hospital, McGill University, Montreal, Quebec, Canada; 7 Department of Clinical and Experimental Medicine and Pharmacology, University of Messina, Messina, Italy; 8 Regional Centre for Drug Evaluation and Information (CREVIF), Department of Pharmacology, University of Bologna, Bologna, Italy; 9 Department of Public Health and Microbiology, University of Turin, Turin, Italy; 10 Center of Epidemiology, Biostatistics, and Medical Information Technology, Polytechnic University of Marche, Ancona, Italy; 11 Department of Public Health and Infectious Diseases, University “La Sapienza”, Rome, Italy; 12 Department of Medicine and Aging, University “G. d’Annunzio”, Chieti-Pescara, Italy

**Keywords:** Bisphosphonates, Drug safety, Healthcare utilization database, Upper gastrointestinal complications

## Abstract

**Background:**

Data on the effect of oral bisphosphonates (BPs) on risk of upper gastrointestinal complications (UGIC) are conflicting. We conducted a large population-based study from a network of Italian healthcare utilization databases aimed to assess the UGIC risk associated with use of BPs in the setting of secondary prevention of osteoporotic fractures.

**Methods:**

A nested case–control study was carried out within a cohort of 68,970 patients aged 45 years or older, who have been hospitalized for osteoporotic fracture from 2003 until 2005. Cases were the 804 patients who experienced hospitalization for UGIC until 2007. Up to 20 controls were randomly selected for each case. Conditional logistic regression model was used to estimate odds ratio (OR) associated with current and past use of BPs (i.e. for drug dispensation within 30 days and over 31 days prior the outcome onset, respectively) after adjusting for several covariates.

**Results:**

Compared with patients who did not use BPs, current and past users had OR (and 95% confidence interval) of 0.86 (0.60 to 1.22) and 1.07 (0.80 to 1.44) respectively. There was no difference in the ORs estimated according with BPs type (alendronate or risedronate) and regimen (daily or weekly), nor with co-therapies and comorbidities.

**Conclusions:**

Further evidence that BPs dispensed for secondary prevention of osteoporotic fractures are not associated with increased risk of severe gastrointestinal complications is supplied from this study. Further research is required to clarify the role BPs and other drugs of co-medication in inducing UGIC.

## Background

Osteoporotic fractures are becoming a major cause of morbidity and mortality worldwide. The lifetime risk of typical osteoporotic fracture (i.e., of the wrist, hip, or vertebra) has been reported to be around 40% [1-3]. Ideally, osteoporosis should be prevented before fragility fractures occur. Nevertheless, an important clinical strategy is to identify patients who have already had a typical osteoporotic fracture and institute treatments aimed at secondary prevention [4-7]. In postmenopausal women, at least 80% to 90% of fractures of the wrist, hip, or vertebra are associated with osteoporosis [8-10] and an osteoporotic patient who experience a fracture has approximately a 20-fold risk of future fracture compared with a patient who has neither osteoporosis nor a history of fracture [2,11].

Oral bisphosphonates (BPs), such as alendronate and risedronate, are considered the mainstay therapy for the prevention of osteoporotic fractures. Randomised clinical trials (RCTs) have consistently shown that long-term treatment with these medicaments improves bone mineral density (BMD) and reduces the risk of fracture [12-19]. However, long-term therapy is required to increase and maintain BMD and to keep normal levels of bone resorption [20]. Therefore, therapy must be generally safe, besides being effective, in a long-term fashion.

Data from the pivotal RCTs of both alendronate [12-14,19] and risedronate [16-18,20,21] did not found clinical evidence of adverse effects than placebo suggesting that these drugs are well tolerated. However, soon after alendronate release, an unexpected higher number of cases of oesophagitis and oesophageal strictures were encountered when the drug was prescribed to the general population, which resulted in changes to the alendronate label [22,23]. From then on nowadays, inconsistent findings on gastrointestinal (GI) safety of BPs have been reported [24-29]. Two meta-analyses on this topic came to conflicting conclusions [30,31], suggesting that evidence on gastrointestinal safety of these agents are still insufficient.

To shed further light on the association between use of BPs and the risk of hospitalization for upper gastrointestinal complications (UGIC), we carried out a large nested case–control study in a cohort of patients hospitalized for osteoporotic fracture.

## Methods

### Data source

The data used for the present study were retrieved from the health service databases of all the 13 Italian territorial units participating at the AIFA-BEST (Bisphosphonates Effectiveness-Safety Tradeoff) project. The general aim of this project is to provide an assessment of the benefit-risk profile of BPs use. Further details of the study design and procedure can be found elsewhere [32].

Territorial units participant to the AIFA-BEST project were four Regions (Abruzzo, Emilia-Romagna, Marche and Toscana) and nine Local Health Authorities (Caserta, Como, Gorizia, Latina, Lodi, Milano, Monza, Sondrio and Varese). A population of about 17 million of beneficiaries of National Health Service (NHS) residents in these territorial units was covered by the corresponding HCU databases, accounting for nearly 30% of the whole Italian population.

Italian population is entirely covered by the NHS that provides universal and free of charge coverage for many healthcares, such as hospitalizations for any causes and several drug therapies (including medicaments for treatment of osteoporosis). This program is administered by an automated system of databases on the use of health services supplied free of charge from NHS and including: (i) an archive of beneficiaries of NHS (practically the whole resident population), inclusive of demographic and administrative data; (ii) details of hospitalizations in private and public hospitals, inclusive of diagnosis at discharge; and (iii) outpatients medicament prescriptions reimbursable from the NHS [according to Italian rules, outpatients medicaments supplied free of charge from NHS may be dispensed only from pharmacies and only by prescription]. With the aim of obtaining the complete history of healthcare utilization of all the NHS beneficiaries, the different pieces of information recorded into these databases can be linked using a unique personal identification code. In order to preserve privacy, we replaced the original identification code with its digest that is the image of the code through a cryptographic hash function -- the Secure Hash Algorithm (SHA-256). Such hash function (i) makes infeasible to obtain the original code from the digest, (ii) is deterministic, i.e. the same digest is always associated to any given individual, and (iii) is collision-resistant, i.e. the probability that two individuals are associated to the same code is insignificant. The specific hash function used (SHA-256) is the industry standard [33] and has been incorporated into the data extraction-transformation-load software produced by the University of Milano-Bicocca.

Data were drawn out from databases by means of standardized queries which were defined and tested according to the study protocol. Additional file 1 provides specific diagnostic and therapeutic codes used for our study.

### Cohort selection

We identified patients aged 45 years or older who have been hospitalized for osteoporotic fracture from July 1, 2003 until December 31, 2005 and the date of hospital discharge was designed as that of entry into the cohort. Patients were excluded if, within six months prior the cohort entry date, they had at least one BPs prescription or they have been hospitalized for bone fracture, gastrointestinal adverse events, Paget’s disease, coagulation disorders, alcohol abuse, chronic liver disease or cancer. Patients who were registered into the archive of NHS beneficiaries from less than six months prior the entry into the cohort and those who did not reach at least 60 days of follow-up were also excluded. The remaining patients constituted the study cohort.

Each member of the cohort accumulated person-years of follow-up from the date of entry until the earliest date among those of outcome onset (hospital admission for UGIC) or censoring (death, emigration or 31 December 2007).

### Selection of cases and controls

We identified patients who during follow-up experienced at least a hospitalization with diagnosis of UGIC including oesophageal/gastrointestinal ulcer, perforation of oesophagus, oesophageal/gastrointestinal haemorrhage (see Additional file 1: Table S1). A patient who experienced at least one of these events was considered as having the outcome. The earliest date of hospital admission recording one of these events was considered as the index date.

Up to twenty controls for each case patient were selected randomly from the cohort to be matched for territorial unit of recruitment, gender, age at cohort entry, date of entry into the cohort and were at risk for the outcome at the time when the matched case had the event. In these conditions each set established from one case and the corresponding matched controls had the same extension of observational period which began at the date of index prescription and stopped at the event date of the index case.

### Exposure assessment

During the study period two drug types (alendronate and risedronate) either on once-daily (10 mg/day and 5 mg/day, respectively) or once-weekly (70 mg/week and 35 mg/week, respectively) regimens were available for free reimbursement by Italian NHS.

Drug-dispensing history of BPs prescribed to cases and controls during the observational period was assessed from the prescription drug database. Exposure was categorized into mutually exclusive groups of current, past, and no use, taking as reference the index date [27]. A patient was defined current user if at least one prescription of BPs was dispensed within 30 days or less prior the index date. Past users were defined as those who at least one prescription of BPs was dispensed later than 31 days prior the index date. No users were patients who during the entire observational period did not experience BPs dispensation.

### Covariates

For each case and control the dispensation of some medicaments over the 60-day period prior the index date was investigated. Medicaments included antidepressants, antithrombotic, gastroprotective agents, corticosteroids, statins, calcium channel blockers, other antihypertensive agents and nonsteroidal antiinflammatory agents (NSAIDs) (see Additional file 1: Table S1). In addition, the Charlson comorbidity index score was calculated [34], using the diagnostic information available from inpatient charts in the six months prior the date of entry into the cohort and during the entire period of follow-up. Two categories of the Charlson comorbidity index score were considered, i.e. 0 and 1, respectively denoting absence and presence of at least one comorbidity.

### Statistical analyses

Chi-square was used to test differences between cases and controls. A conditional logistic regression model was fitted to estimate the odds ratio (OR), as well as its 95% confidence interval (CI), of UGIC associated with use of BPs (anytime, current or past), taking non-use as reference. Adjustments were made for the above reported covariates. The combined effect of BPs with co-treatments and co-morbidity was estimated by including the corresponding interaction terms in a conditional logistic model. The differential effect of type and regimen of the dispensed BPs was also evaluated by means of stratification analysis.

### Sensitivity analyses

The following sets of sensitivity analyses were performed. First, we verified if our estimates were affected by the adopted criteria for defining UGIC. Data were analysed according to alternative diagnostic criteria, i.e. those recently proposed by *Cadarette* et al. while investigating oral BPs safety [28], as well as those used by a collaborative project aimed to exploit European healthcare databases for drug safety signal detection, the so called EU-ADR Project [35]. Second, we verified if our estimates were affected by the adopted criteria for defining exposure. With this aim we used time-window lengths of 7, 15 or 45 days prior the index date for defining current use, alternative to 30 days as in the main analysis.

The SAS statistical package was used for the analyses (SAS, Version 9.1; SAS Institute, Cary, North Carolina, USA). For all hypotheses tested two-tailed p-values less than 0.05 were considered to be significant.

### Ethical considerations

The study protocol was notified to the Italian Medicines Agency (AIFA) and to the local ethics committees of all the territorial units involved in the investigation. There was no legal requirement for ethics committee approval since we used only unidentifiable patient data and did not contact the patients.

## Results

### Sample selection

**Figure 1:**
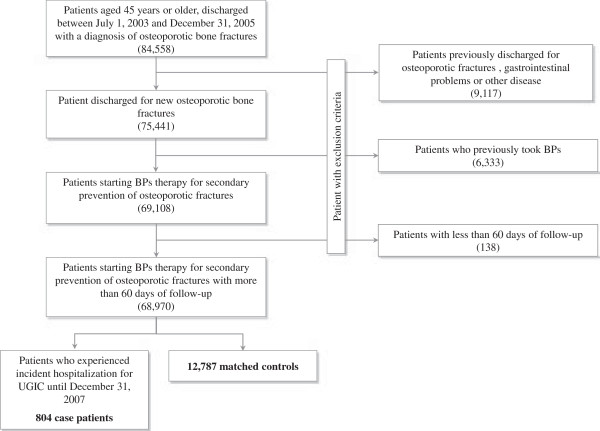
**Study flow diagram.** AIFA-BEST Project, Italy, 2003–2007. Flow chart of inclusion and exclusion criteria. BPs: Bisphosphonates.

The distribution of the exclusion criteria is shown in Figure 1. At entry, the 68,970 patients who were included into the cohort had mean age of 76.2 years (SD 12.5 years) and 71% of them were women. During follow-up these patients accumulated 220,135 person-years of observation and generated 804 hospital admissions for UGIC, with an incidence rate of 36.5 cases per 10,000 person-years. The 804 patients who experienced hospitalization for UGIC (case patients) were matched to 12,787 controls.

### Patients

At the cohort entry, mean age of cases and controls was 79.9 years (SD: 9.9 years), and nearly 72% of them were women (matching variables). As shown in Table 1, there was not statistical evidence that case patients and controls differed for use to BPs during the entire observational period, as well as during current and past periods. Similarly, there was not evidence that cases and controls differ for BPs type and regimen refilled during the current period. Conversely, with the exception of statins and calcium channel blockers, co-treatments with the other considered drugs, as well as the presence of at least one sign of chronic comorbidity, were more frequent among cases than controls.

**Table 1:** Selected tracts of the 804 cases of upper gastrointestinal complications and 12,787 controls

	**Case patients**	**Controls**	p-value^*^
**BPs exposure**^**†**^			
No use	709 (88.2%)	11,345 (88.7%)	0.6029
Current use	38 (4.7%)	643 (5.0%)	
Past use	57 (7.1%)	799 (6.2%)	
Type prescribed during the current period			
Alendronate	30 (79.0%)	412 (64.1%)	0.0620
Risedronate	8 (21.0%)	231 (35.9%)	
Regimen prescribed during the current period			
Weekly	37 (97.4%)	631 (98.1%)	0.7376
Daily	1 (2.6%)	12 (1.9%)	
**Use of other medicaments**^**‡**^			
Antidepressants	139 (17.3%)	1,841 (14.4%)	0.0242
Antithrombotic	240 (29.9%)	3,174 (24.8%)	0.0014
Gastroprotective agents	211 (26.2%)	1,993 (15.6%)	<0.0001
Corticosteroids	65 (8.1%)	533 (4.2%)	<0.0001
Statins	41 (5.1%)	724 (5.7%)	0.5021
Calcium channel blockers	105 (13.1%)	1,548 (12.1%)	0.4223
Other antihypertensive drugs	371 (46.1%)	5,294 (41.4%)	0.0082
Nonsteroidal antiinflammatory drugs	170 (21.1%)	1,529 (12.0%)	<0.0001
**Co-morbidity**^**#**^			
0	629 (78.2%)	11,531 (90.2%)	<0.0001
≥1	175 (21.8%)	1,256 (9.8%)	

BPs: Bisphosphonates.

^†^A patient was defined current user if at least one prescription of BPs was refilled within 30 days or less prior the index date. A patient was defined past user if at least one prescription of BPs was refilled later than 31 days prior the index date. No users were patients who during the entire observational period did not experience BPs dispensation.

^‡^Measured over the 60-day period prior the index date.

^#^Charlson comorbidity index score measured in the six months prior the entry into the cohort and during the entire observational period.

^*^According to chi-square test.

### Use of bisphosphonates and the risk of upper gastrointestinal complications

**Figure 2:**
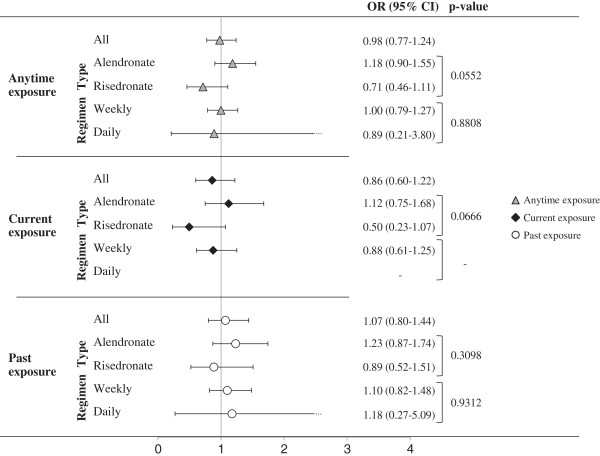
**Adjusted odds ratios (and 95% confidence intervals) of upper gastrointestinal complications associated with anytime, current and past exposure to bisphosphonates as a whole (all) as well as to type (alendronate and risedronate) and regimen (daily and weekly) of the latest dispensed bisphosphonates.** AIFA-BEST Project, Italy, 2003–2007. Odds ratios estimated with conditional logistic regression model. Estimates concerning main analysis (all) were adjusted for use of other medicaments in the 60-day period and for the Charlson index measured before the index date. Estimates concerning subgroup analysis were obtained by including the interaction terms combining the effect of anytime, current or past exposure to BPs together with type and regimen of the dispensed BPs. P-values concern comparison of BPs effect across patient subgroups. BPs: Bisphosphonates.

Compared with non users, patients who used BPs anytime during the entire observational period, as well as those who were exposed during current or past period, did not show significant risk excess of UGIC (Figure 2). In addition, there was no evidence that UGIC risk was heterogeneous across the categories of both types and regimens of BPs refilled anytime during the observational period, nor during current and past periods.

**Figure 3:**
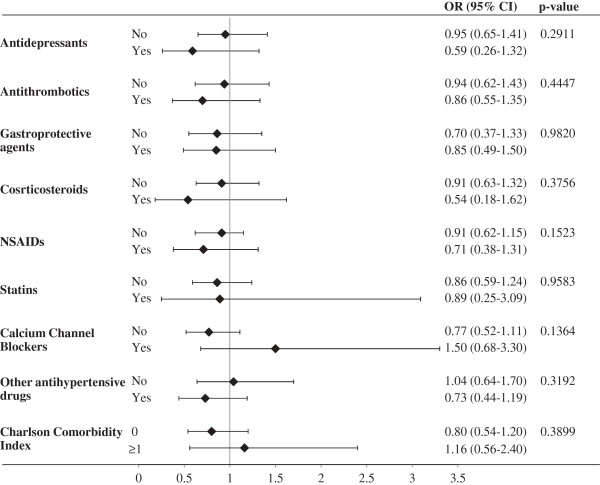
**Combined action of current exposure to bisphosphonates, concurrent exposure to other medicaments and categories of Charlson comorbidity index on the risk of upper gastrointestinal complications.** AIFA-BEST Project, Italy, 2003–2007. Odds ratios estimated with conditional logistic regression model. Estimates were obtained by including the interaction terms combining the effect of current exposure to BPs together with concurrent use of other medicaments and the categories of the Charlson index. P-values concern comparison of BPs effect across patient subgroups. BPs: Bisphosphonates.

As shown in Figure 3, there was not statistical evidence that the UGIC risk associated with current use of BPs was heterogeneous across the categories of patients stratified according with co-treatments and comorbidity. It should be observed, however, that large confidence intervals were obtained for some strata. This was due to the few patients who concomitantly used BPs and other medicaments such as corticosteroids, statins or calcium channel blockers (being the corresponding prevalence 8.6%, 9.3% and 13.3% respectively).

### Sensitivity analyses

**Figure 4:**
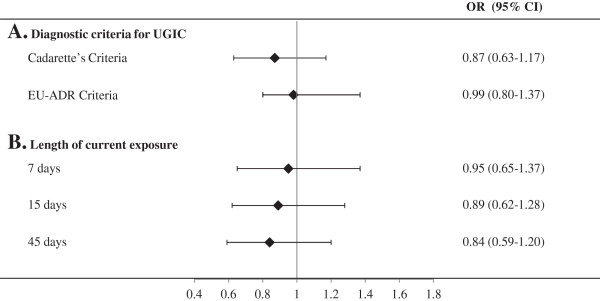
**Influences of diagnostic criteria for defining upper gastrointestinal complications (panel A), and of the time-window length for defining current use of BPs (panel B) on the observed odds ratio of upper gastrointestinal complications associated with current exposure to bisphosphonates.** AIFA-BEST Project, Italy, 2003–2007. Odds ratios estimated with conditional logistic regression model. Estimates were adjusted for use of medicaments in the 60-day period and for the Charlson index measured before the index date. Details for diagnostic criteria are reported in Additional file 1. BPs: Bisphosphonate.

Figure 4 shows that the adjusted OR did not substantially change by varying criteria for defining diagnosis of UGIC (box A) nor the length of exposure time-window for defining current use of BPs (box B).

## Discussion

In this large nested case–control study we did not found evidence of increased severe UGIC risk associated with current and/or past use of BPs in the setting of secondary prevention of osteoporotic fractures. We also found that the type and regimen of BPs administered, as well as the concurrent use of other drugs known for increasing UGIC, did not modify the gastrointestinal safety of BPs.

### Comparison with literature

Our results are consistent with those of two randomized clinical trials (RCTs), FIT (Fracture Intervention Trial) and VERT (Vertebral Efficacy of Risedronate Therapy) trials [16,36], both reporting similar gastrointestinal side-effects profile in patients who received BPs and those on placebo. Similarly to ours, a pooled analysis from 9 RCTs found that the rate of upper GI tract adverse events was similar across risedronate and placebo groups, and that concomitant use of aspirin, NSAIDs, H_2_-receptor antagonists and/or proton pump inhibitors did not lead to significant between-group differences in the UGIC risk [37].

Validity of our main findings seems to have support by the observed association between use of other medicaments known to cause GI complications and the considered outcome. For example, consistently with literature, we found that, with respect to controls, case patients had higher prevalence in the use of antidepressants, antithrombotic, corticosteroids, some class of antihypertensive agents, and NSAIDs, in the presence of chronic comorbidities [38-45]. Conversely to others, we did not confirm that recent use of calcium-channel blockers and statins increased the risk of UGIC [45,46].

We found that cases had higher use of gastroprotective drugs than controls. As we cannot suppose that gastroprotective agents cause GI complications, the more likely explanation is that physicians more likely prescribe gastroprotective agents to those patients with a history of GI complications, or to those at whom GI symptoms sudden occurred, i.e. to patients at higher UGIC risk [47].

### Strengths and limitations

Several peculiar features of our study deserve to be mentioned. First, the study is based on data from a very large unselected population, which was made possible by the fact that in Italy a cost-free uniformly organized healthcare system involves practically all citizens. By drawing out healthcare utilization data from nearly 30% of the whole Italian population, we were able to build one of the largest observational studies performed on the GI safety of bisphosphonates in the setting of secondary prevention of osteoporotic fractures. Accordingly with the included number of cases and controls, as well as of the observed number of controls who currently used BPs, even small gastrointestinal effects associated with current use of BPs (ORs ≥ 1.5) could have been detected from our study as significant (with p < 0.05 and power of 80%). Our data, furthermore, reflecting routine clinical practice, are unaffected by selective participation and recall bias.

Second, the drug prescription database provided highly accurate data, because report of prescriptions by the pharmacies is essential for reimbursement and filling of an incorrect report about the dispensed drugs has legal consequences [48]. Third, a number of sensitivity analyses allowed us to verify the robustness of our findings. For example, we found that the criteria employed for UGIC diagnosis, as well as for defining current BPs use, did not substantially affect our estimates. This further strengthens the evidence that the use of BPs unlikely causes serious GI complications.

Our study has a number of potential limitations. First, as outcomes were drawn from hospitalized patients, our data only concern severe GI complications requiring hospitalization. Second, because of privacy regulations, hospital records were not available for analysis so diagnoses cannot be scrutinized and validated. Third, misclassification of BPs exposure might occur because, once the drug is dispensed, it is possible that patients did not assume it. If this happens when GI symptoms occur, a protopathic bias is introduced dragging the investigated association towards that expected under the null hypothesis [49].

Fourth, besides the very large sample size, some of our findings are likely to be affected by random uncertainty. For example, during the study period BPs were rarely dispensed once-daily, so the lack of evidence of differential effect of BPs according to the dispensation regimen is particularly weak. Similarly, the lack of evidence of a synergistic effect between BPs and other known GI-harmful drugs might be due to the few number of patients taking co-therapies during the current period.

The uncommon dispensation of BPs and other medicaments observed in our setting, however, has important clinical implications. For example, patients with more severe form of osteoporosis may need to assume antiinflammatory agents (e.g. NSAIDs or corticosteroids) for the symptomatic relief of pain secondary to this disease. In this way, a high prevalence of patients who concomitantly use BPs and antiinflammatory agents are expected in routine clinical practice. Since BPs co-therapy with NSAIDs has been found to increase the risk of peptic ulcer in rheumatoid arthritis patients with long-term NSAIDs treatment [30], this practice should be avoided, as often occurs in our setting.

Finally, as in all observational studies, there is always some concern for residual confounding due to unmeasured factors. For example, under the assumption that use of BPs increases the UGIC risk, over-the-counter gastroprotective agents might be assumed by some patients once GI symptoms occur. On the other hand, the assumption of over-the-counter antiinflammatory agents might be reduced when GI symptoms occur. Both these sources of selective exposures would drag the investigated association towards that expected under the null hypothesis.

## Conclusions

In summary this study provides further evidence that BPs dispensed for secondary prevention of osteoporotic fractures are not associated with increased risk of severe gastrointestinal complications. Further research is required to clarify the role of co-medication with BPs and other medicaments in inducing upper gastrointestinal complications.

## Competing interests

The authors declare that they have no competing interests.

## Authors’ contributions

AG and LS performed the statistical analyses, GDV was responsible for producing software assuring privacy, AG and GC wrote the paper, GC was responsible for designing the current study, GM was the main investigator of the AIFA-BEST project, LCDO, FL, FC, AC, AV, DG, RG, ARV and TS were the other investigators of the AIFA-BEST; they participated to the project’s design and supplied data. All authors read and approved the final manuscript.

## Pre-publication history

The pre-publication history for this paper can be accessed here:

http://www.biomedcentral.com/1471-230X/14/5/prepub

## Supplementary Material

Additional file 1 **Drugs and diagnoses codes used for the study purpose**[28,35]. Click here for file

## Best investigators

Mazzaglia G, (main investigator): Regional Agency for Healthcare Services of Tuscany, Italy

Cipriani F (Head Unit), Lapi F, Sessa E: Regional Agency for Healthcare Services of Tuscany, Italy

Caputi AP (Head Unit), Arcoraci V: Department of Medicine and Pharmacology, University of Messina, Messina, Italy

Corrao G (Head Unit), Ghirardi A, Scotti L, Parodi A, Zambon A: Department of Statistics and quantitative methods, Unit of Biostatistics, epidemiology and public health, University of Milano-Bicocca, Milan, Italy

Montanaro N (Head Unit), Piccinni C, Suzzi C, Puccini A, Vaccheri A: Regional Centre for Drug Evaluation and Information (CREVIF), Department of Pharmacology, University of Bologna, Bologna, Italy.

Sturkenboom M (Head Unit): Departments of Epidemiology & Biostatistics and Medical Informatics, Pharmacoepidemiology Unit, Erasmus University Medical Center, The Netherlands

Geppetti P (Head Unit), Sati L, Di Bari M: Center of Pharmacoutilization, Pharmacoepidemiology, Pharmacovigilance and Pharmacoeconomics, University of Florence, Florence, Italy

Gregori D (Head Unit), Forlan F: Unit of Biostatistics, Epidemiology and Public Health, Department of Cardiac, Thoracic and Vascular Sciences, University of Padova

Carle F (Head Unit), Gesuita R: Center of Epidemiology, Biostatistics, and Medical Information Technology, Polytechnic University of Marche, Ancona, Italy. Provided data from Marche

Staniscia T (Head Unit), Menna A: Regional Agency of Healthcare services of Abruzzo, L’Aquila, Italy

Vestri A (Head Unit): Department of Public Health and Infectious Diseases, University “La Sapienza”, Rome, Italy

Valenti M (Head Unit): Department of Clinical Sciences and Applied Biotechnology, University of L’Aquila, L’Aquila, Italy
